# Grasping nettles: cellular heterogeneity and other confounders in epigenome-wide association studies

**DOI:** 10.1093/hmg/ddu284

**Published:** 2014-06-13

**Authors:** Liming Liang, William O.C. Cookson

**Affiliations:** 1 Department of Epidemiology and; 2 Department of Biostatistics, Harvard School of Public Health, Boston, MA 02115, USA and; 3 National Heart and Lung Institute, Imperial College London, London SW3 6LY, UK

## Abstract

Platform technologies for measurement of CpG methylation at multiple loci across the genome have made ambitious epigenome-wide association studies affordable and practicable. In contrast to genetic studies, which estimate the effects of structural changes in DNA, and transcriptomic studies, which measure genomic outputs, epigenetic studies can access states of regulation of genome function in particular cells and in response to specific stimuli. Although many factors complicate the interpretation of epigenetic variation in human disease, cell-specific methylation patterns and the cellular heterogeneity present in peripheral blood and tissue biopsies are anticipated to cause the most problems. In this review, we suggest that the difficulties may be exaggerated and we explore how cellular heterogeneity may be embraced with appropriate study designs and analytical tools. We further suggest that systematic mapping of the loci influenced by age, sex and genetic polymorphisms will bring important biological insights as well as improved control of epigenome-wide association studies.

## INTRODUCTION

Epigenetics broadly defines the study of changes that affect DNA function without altering the nucleotide sequence. Epigenetics is now a hot area in medical genomics. Large-scale projects such as the International Human Epigenome Consortium (http://ihec-epigenomes.org/) are generating comprehensive measurements of the variation in DNA methylation, histone modification, nucleosome occupancy and coding and non-coding RNA expression from normal and diseased cells. These data intersect with the ENCyclopedia Of DNA Elements (ENCODE) project that is building a comprehensive list of the functional elements of the human genome by studying >4000 different datasets covering a wide range of cells, conditions and technologies (http://genome.ucsc.edu/ENCODE/index.html).

These resources offer wonderful opportunities for understanding and mapping genome function in health and disease. Although there are many epigenetic markers that may be of use in systematic studies, the degree of CpG methylation at multiple sites (loci) across the genome is at the moment most easily applicable to understanding disease. Hypermethylation in regulatory regions such as CpG islands, which are often associated with transcription start sites, are generally associated with genes that are not expressed, although other CpG sites in untranscribed genes may exhibit hypomethylation (1).

It is not yet clear to what extent methylation in regulatory regions of genes is directly modifying gene expression or function and how much is the consequence of the binding of classical transcription and enhancer factors. Nevertheless, it is generally true that genome-wide methylation assays capture robust biological information about the functional state of cells and tissues. It is reasonable to expect that this information can be used to detect novel genes and pathways underlying disease and to develop systematic understanding of essential processes such as inflammation and repair.

The effective use of genome-wide data of this extent and complexity is however demanding. Known but relatively uncharted effects on epigenetic marks at multiple sites include age, sex, the environment and DNA variants such as Single Nucleotide Polymorphisms (SNPs). Investigators will also have to deal with batch effects that may arise from the platforms used to quantify epigenetic variation.

A current perception is that the major problem with epigenome-wide studies arises because epigenetic changes determine (or reflect) cellular differentiation into specific lineages (2–4), requiring purified cells to be studied (5) or that the cell-specific patterns be evaluated before attempting association studies (6). If true, given that most tissues are complex mixtures of different cells in varying stages of activity, then progress will be slow. Happily, there are ways to deal with cellular heterogeneity that do not involve exhaustive isolation of every cell type.

The main purpose of this review is to explore practical and analytical approaches to CpG methylation that may be used to control for cellular mixtures, whilst taking into account other major potential confounders of epigenome-wide surveys.

## EXPERIMENTAL DESIGN: EXERCISING THE GENOME

A full discussion of experimental design is beyond the remit of this review, but the concept of expression space of an individual genome is worth considering in strategies to extract the maximum information from any study (7). The expression space (or the epigenomic space) of a genome represents the spectrum of various functions that the genome may carry out. Exercising the genome captures states that are relevant to the question under study. Experiments that exercise the epigenome may examine the genome at different points in time after a stimulus, or at different concentrations of a stimulus (and considering whether the stimulus is physiological or pharmacological), or testing in different subjects with different genetic and epigenetic backgrounds and examining genome function in different cells and tissues (7). To this list can be added environmental and disease-related stimuli that may strongly influence the epigenome.

Most epigenome-wide association studies will be based on a cross-sectional observational design that is similar to that applied to genetic association studies. However, even this simple structure may draw on the epigenomic space in unexpected ways.

Whilst DNA sequence changes are stable over the lifetime of an individual, epigenetic changes may represent dynamic responses to known and unknown confounding factors, such as age, sex, the environment and disease that may need to be considered in study design.

If these factors confound the outcome or exposure of interest in a cross-sectional study, they can inflate false-positive rates as well as reduce statistical power to capture real associations. In common with gene expression studies and expression quantitative loci (eQTL) mapping, it may be anticipated that statistical power will be increased by modelling unknown as well as known factors in association studies (8). We consider these below.

## BATCH AND PLATFORM EFFECTS

The technology for genome-wide measures of CpG methylation is steadily evolving. Illumina provides a popular platform for the robust measurement of methylation status at 450 000 CpG residues across the genome, with matching probes comparing sequences for native DNA compared with sequences after bisulphite conversion. This technology is likely to be replaced by whole-genome bisulphite sequencing, with or without a means for enriching for loci showing significant variation between cells, tissues and individuals (9) [it is worth noting that other cytosine modifications such as 5-hydroxymethylcytosine and 5-formylcytosine are found in genomic DNA of a wide range of mammalian cells and may carry additional information (10)]. The outcome of these measurements is a quantitative parameter that captures the degree of methylation at a particular site [in the case of Illumina platform, the parameter is β, measured on a scale of 0 (completely unmethylated) to 1 (completely methylated)].

As with any microarray platform that measures quantitative traits, batch effects, such as those associated with chips, plates, runs, time and other experimental and biological conditions, are expected to cause a large variation in parameter measurement. Adjusting effectively for these can increase power and reduce false positives. Adjustment can be accomplished by treating batch ID as a factor variable included as a fixed effect in the regression model to test association. When the sample size is small, an empirical Bayes method can be used to estimate batch effects jointly using all probes on the array (11). An alternative is to model the batch ID as a random intercept in the regression model, saving degrees of freedom and potentially increasing power.

Confounding factors such as environmental exposures and technical variations may commonly be represented in the array data without being known or observable. High dimension methods such as principal component analyses (PCA) and multidimensional scaling (MDS) can be used effectively to estimate and adjust for such factors when they are sufficiently strong (12,13). Similar adjustments have provided marked improvements in power to detect eQTL associations in genome-wide surveys (8,14).

## AGE AND SEX

The age of subjects is known to influence methylation at loci across the genome. Although monozygotic twins may be indistinguishable by methylation status at birth, consistent age-related changes (both negative and positive) may be detected as children grow older (15). Gene ontology analyses indicate that these loci affect genes for developmental processes and immune functions and that there is overlap with loci that change with age in adult studies (15). In healthy adults (post-menopausal women), most age-related changes involve locus hypermethylation and are not associated with the disabilities that variably accompany ageing (16).

In contrast, other studies of adults suggest enrichment for structural motifs such as bivalent chromatin domains in age-associated DNA hypermethylation (17) and that these changes are not immune or haemopoietic cell-type specific (17). It has also been suggested that polycomb group proteins, which are supressed in stem cells, may show increased methylation in post-menopausal women, predisposing to malignancy (18). Other authors have opined that genome-wide methylation profiles may be usefully employed to quantify human rates of aging (19).

These papers reflect different schools of interpretation of genome-wide methylation signals, the best of which aims for reproducibility, meta-analyses, and mapped and confirmed loci that eventually can be functionally interrogated. Importantly, at least some of the age-related changes in methylation at specific loci may be attributed with varying composition of different white cells in the peripheral blood leukocytes (PBLs) that are the source of DNA for many genetic and epigenetic association studies (20). These difficulties arising from cellular heterogeneity are discussed in detail below.

The effects of sex on methylation status are well documented (21,22), beginning with the recognition that X-chromosome inactivation in females is accompanied by widespread CpG hypermethylation (23). Interestingly, the pattern of genes showing methylation on the X chromosome is tightly regulated and variable between individuals (24) may even be tissue specific (25). Sex-related changes in methylation are also recognized at autosomal loci (21,22), but these effects have not yet been systematically mapped or studied. A further complexity arises from the recognition that environmental effects may produce different methylation consequences in males and females, for example genes influencing glucose metabolism, obesity and diabetes amongst neonates in rural Gambia (26).

## GENETIC POLYMORPHISM

Genetic polymorphisms such as SNPs are recognized to affect methylation at individual loci (27) and genome wide (28,29). These effects are strongest in *cis* but are also present in *trans*. The mechanisms for SNP effects are not well understood but are likely to relate to allelic differences in the binding or expression of regulatory factors, including non-coding RNAs.

True SNP effects need to be differentiated from genetic variants that underlie the methylation probe sequence or the CpG interrogation site. These polymorphisms can cause confounding between methylation and the outcome of interest, as the perceived association with methylation is in fact the association with genotypes of the genetic variant. Such methylation probes are easily removed during quality control steps of analysis.

Genetic variants may confound associations even when the probe sequence or the CpG site does not overlap with any polymorphism. A genetic variant can be a regulator of the disease outcome at the same time as influencing methylation at a CpG site. In this case, association between outcome and methylation reflects sharing of genetic controls rather than causality. One can use mediation analysis (30–33) to estimate how much SNP–outcome association is through methylation as an intermediate phenotype and Mendelian randomization (34) to assess the causal direction between SNP, methylation and outcome.

## CELL-SPECIFIC EFFECTS

Lineage commitment to particular cell types can be identified by methylation status at specific loci (2–4). There has been considerable concern that studying heterogeneous mixtures of cells from tissues and peripheral blood will cause serious confounding of epigenome-wide association studies (5,6) and that as a consequence purified cells should be studied (5) or that the cell-specific pattern for the gene region of interest should be evaluated before attempting association studies (6).

If this assumption is correct, then larger-scale epigenome studies will prove very technically difficult to perform. It is worth observing that cell specificity applies equally to studies of global gene expression, and, despite the old canard that gene expression in peripheral blood measured by microarrays was really an expensive cell count, there have been enormous advances in mapping eQTLs from lymphoblastoid cell lines and tissues with heterogeneous composition (8,35,36). Such studies have been particularly helpful in understanding systematically the function of the numerous disease-associated loci discovered by genome-wide association studies (GWAS) (35).

Before exploring means of resolving or controlling for cellular heterogeneity, there are two further complicating factors that are not usually considered. First, not all PBL constituents will be treated equally by the process of DNA extraction: buffy coats are retrieved after centrifugation that separates some granulocytes (such as eosinophils and basophils) from lymphocytes, monocytes and neutrophils, and red cell lysis methods may have differential effects on monocytes compared with other PBLs. It is therefore essential that cases and controls in a study have DNA extracted by matching protocols and that the results from different subjects and panels are amalgamated by meta-analyses rather than simple pooling.

**Figure 1.**
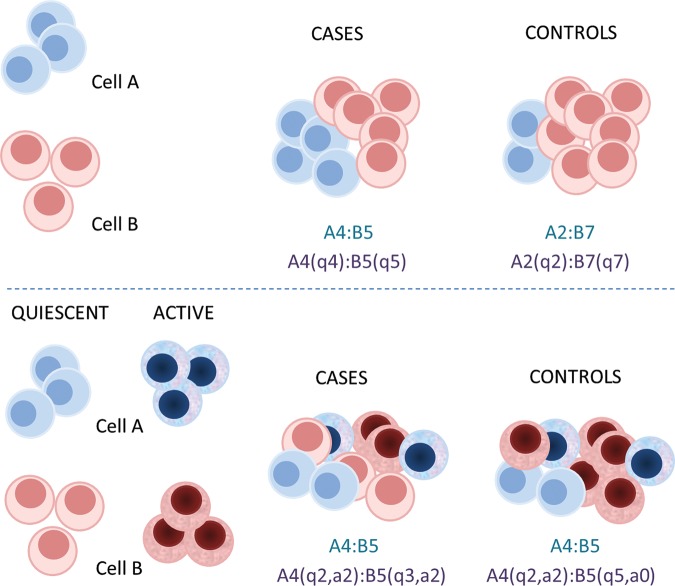
Cellular heterogeneity and epigenetic association. The top panel of the figure shows how two types of cells (**A** and **B**) may be in different proportions in tissue samples from cases (for example, A4 : B5) and controls (A2 : B7). If epigenetic variation between cases and controls is confined to these cells, then their proportions (shown in blue) will be reflected in their epigenetic ratios (shown in purple: **q** indicates the number of cells that are quiescent). This does not, however, render the results of association testing unimportant. The lower panel illustrates the case when cell type A and cell type B are capable of quiescent and active states (as, for example, is usual for immunological cells). Here, simple cell counts will miss significant alterations in the epigenetic profile of cell type B (shown in purple: **q** indicates the number of cells that are quiescent and **a** the number that are active). In both examples, the measure of epigenetic changes provides an accurate estimate of differences between case and control samples.

Secondly, and equally of relevance, most PBLs exist in activated and unactivated states, including neutrophils (37), eosinophils (38,39), monocyte–macrophages (40) and T cells and B cells (41). Based on knowledge of gene expression, activation will in each case be accompanied by important changes in methylation profiles (Fig. 1). The manipulation of cells during their purification may also perturb their methylation profile and gene expression. In these circumstances, simplistic isolation of pure cell types and their use as references may generate additional ungovernable influences on study outcomes.

Will it then be possible to detect meaningful associations from unfractionated cells? Assuming experimental variables (ancestry, DNA extraction methods, batch and platform effects) are controlled, then the power to detect cell-specific associations from PBLs and other tissues will depend on the proportion of each cell type, the effect size in specific cells and the sample size (Fig. 1). Assuming the variance at a locus arises from a particular cell type in a sample, then the overall variance in DNA from PBL or a tissue will produce an attenuation of the effect size in specific cells that would mask associations rather than magnify them. Similarly, the study of the wrong cell types (such as in some circumstances, the Epstein–Barr virus-transformed lymphoblastoid cell lines that are commonly used as a renewable source of DNA for genetic studies) will produce null results rather than systematic false positives.

Pushing on and ignoring heterogeneity for the moment, a positive association result may then be entirely due to the presence of particular cells or to the activation of particular cells, or as is perhaps most likely, a combination of these effects (Fig. 1) (it is also quite conceivable that the effects may be general and not cell specific at all). These primary results may themselves be of interest, such as the highly reproducible effects of cigarette smoking on genes potentially affecting coagulation pathways (42,43), but a general next step becomes to engage cellular heterogeneity and to differentiate between possibilities.

Although whole blood is often the only available tissue in large-scale epidemiology studies, it is fortunate that phenotyping of subjects often includes full blood counts (FBCs, also complete blood counts), which are routinely measured by clinically standardized automated procedures. The FBC gives absolute counts and proportions of all major constituents of PBL, with high reproducibility and well-understood normal values.

To account for cellular heterogeneity in epigenetic association studies, one commonly used approach is to include these white cell counts (or their proportions) as fixed effects in regression models (44). These factors are often included as linear terms in the regression model and can adjust for confounding in most situations, although non-linear effects from cell counts should be considered when examining interactions (45).

In the absence of an FBC, or for other factors that are known but not observed, association models can in theory be built on factors that are estimated from known methylation patterns in particular cell types such as CD8+, CD4+ and natural killer T cells, based on a cell-sorted methylation data (5,20). These achieve reliable estimates of fractions of major cell types in tightly controlled experiments, but their practical utility may be confounded by disease-relevant cellular activation in patients and population samples (Fig. 1). Additionally, reference panels based on sorted cells are not available from important sources such as adipose or tumour tissue.

In the absence of reference data, major components in genome-wide DNA methylation patterns from PBL can be estimated and used as surrogates of cell proportions (46,47), even to the suggested extent that it may be helpful in non-hematopoietic cancers (48).

Strong associations between methylation and exposure or outcome should be taken into account when estimating surrogate components, using analyses such as those implemented in RefFreeEWAS (49), surrogate variable analysis (50) in SVA (12) or SVA-PLS (51) and the Bayesian factor analysis package PEER (13,52). Surrogate components, estimated explicitly with PCA or MDS or implicitly using linear mixed models such as EWASher (53), may lose power to detect true association with large effect sizes but are of most use when effect size is smaller than confounding factors (8,14). Genomic control remains a final approach to correct for systematic inflation in false positives in an association study (54).

A further approach to detecting for cell-specific effects may lie in network analyses, which can identify co-regulated gene modules that represent functional biological units of a system (55). Correlation-based networks discovered through the WCGNA package (56) have previously been shown to strongly correlate with particular cell types using global gene expression (57,58) as well as global CpG methylation patterns (59).

Whilst certainly helpful, it is likely that the statistical inferences described earlier will never be better than direct cell counts, and any models incorporating measured or imputed cell counts may never be completely convincing, at least to referees.

For epigenetic associations to be substantiated, the onus still falls on validation of primary associations in secondary panels of subjects, ideally followed by the demonstration of effects in isolated cells from cases and controls. In understanding the functional consequences of epigenetic associations, it may become important to appreciate that methylation shows a complex and currently unpredictable relationship to gene expression (60).

## CONCLUSIONS

The use of epigenome-wide information in association with other studies is therefore far from facile. In addition to effects of age, sex, genetic polymorphism, cellular heterogeneity and the environment are added DNA extraction methods, batch effects and the recognition that activated immune, and other cells exhibit a very different epigenotype to their resting namesakes. Nevertheless, these obstacles are surmountable with appropriate study designs and analytical tools. We believe that systematic mapping of loci influenced by age, sex and SNPs will allow direct control of their influence in future studies, as well as examining important biological processes.

Finally, it should be realized that dynamic epigenetic changes may follow rather than initiate disease processes, so that association with epigenetic variation may not indicate causality in the same way as an SNP association. Nevertheless, epigenomics provides the opportunity for remarkable insights into genome function. Global gene expression is of proven value in interpreting the functional consequence of disease associations (36), but its measurement is expensive and requires stringent sample collection and storage. In contrast, DNA from PBL is often available from historic population and case–control studies, so that genome-wide mapping of CpG methylation may directly inform on mechanisms of diverse diseases, even from DNA that is 30 000 years old (61).

## FUNDING

The Freemasons’ Grand Charity, the Wellcome Trust under WT 077959 and WT096964 and the NIH R01 HL101251–01. Funding to pay the Open Access publication charges for this article was provided by The Wellcome Trust.

*Conflict of Interest statement*. None declared.
